# Risk factors for 14-day rehospitalization following trauma with new traumatic spinal cord injury diagnosis: A 10-year nationwide study in Taiwan

**DOI:** 10.1371/journal.pone.0184253

**Published:** 2017-09-01

**Authors:** Carlos Lam, Ping-Ling Chen, Jiunn-Horng Kang, Kuang-Fu Cheng, Ray-Jade Chen, Kuo-Sheng Hung

**Affiliations:** 1 Emergency Department, Department of Emergency and Critical Care Medicine, Wan Fang Hospital, Taipei Medical University, Taipei, Taiwan; 2 Graduate Institute of Injury Prevention and Control, College of Public Health, Taipei Medical University, Taipei, Taiwan; 3 Department of Emergency Medicine, School of Medicine, College of Medicine, Taipei Medical University, Taipei, Taiwan; 4 Department of Physical Medicine and Rehabilitation, Taipei Medical University Hospital, Taipei Medical University, Taipei, Taiwan; 5 Department of Physical Medicine and Rehabilitation, School of Medicine, College of Medicine, Taipei Medical University, Taipei, Taiwan; 6 Biostatistics Center, College of Management, Taipei Medical University, Taipei, Taiwan; 7 Department of Surgery, School of Medicine, College of Medicine, Taipei Medical University, Taipei, Taiwan; 8 Division of General Surgery, Department of Surgery, Taipei Medical University Hospital, Taipei, Taiwan; 9 Department of Neurosurgery, Wan Fang Hospital, Taipei Medical University, Taipei, Taiwan; University of Toronto, CANADA

## Abstract

**Objectives:**

Fourteen-day rehospitalization with new traumatic spinal cord injury (tSCI) diagnosis is used as an indicator for the diagnostic quality of the first hospitalization. In this nationwide population-based cohort study, we identified risk factors for this indicator.

**Methods:**

We conducted a nested case–control study by using the data of patients who received a first hospitalization for trauma between 2001 and 2011. The data were retrieved from Taiwan’s National Health Insurance Research Database. Variables including demographic and trauma characteristics were compared between patients diagnosed with tSCI at the first hospitalization and those receiving a 14-day rehospitalization with new tSCI diagnosis.

**Results:**

Of the 23 617 tSCI patients, 997 had 14-day rehospitalization with new tSCI diagnosis (incidence rate, 4.22%). The risk of 14-day rehospitalization with new tSCI diagnosis was significantly lower in patients with severe (injury severity score [ISS] = 16–24; odds ratio [OR], 0.17; 95% confidence interval [CI], 0.13–0.21) and profound (ISS > 24; OR, 0.11; 95% CI, 0.07–0.18) injuries. Interhospital transfer (OR, 8.20; 95% CI, 6.48–10.38) was a significant risk factor, along with injuries at the thoracic (OR, 1.62; 95% CI, 1.21–2.18), lumbar (OR, 1.30; 95% CI, 1.02–1.65), and multiple (OR, 3.23; 95% CI, 1.86–5.61) levels. Brain (OR, 2.82), chest (OR, 2.99), and abdominal (OR, 2.74) injuries were also identified as risk factors. In addition, the risk was higher in patients treated at the orthopedic department (OR, 2.26; 95% CI, 1.78–2.87) and those of other surgical disciplines (OR, 1.89; 95% CI, 1.57–2.28) than in those treated at the neurosurgery department.

**Conclusions:**

Delayed tSCI diagnoses are not uncommon, particularly among trauma patients with ISSs < 16 or those who are transferred from lower-level hospitals. Further validation and implementation of evidence-based decision rules is essential for improving the diagnostic quality of traumatic thoracolumbar SCI.

## Introduction

Time is a critical factor for preserving residual neurological function and preventing secondary injury after traumatic spinal cord injury (tSCI) [1–3]. Prompt recognition, protection, and transport of victims to a facility capable of providing definitive diagnosis and treatment are considered the current standards for the acute treatment of tSCI patients [4, 5]. However, an inappropriate delay in diagnosing tSCI is still reported among trauma patients, and increases the risk of permanent sequelae for patients and that of medical-legal liability for treating surgeons [6–8].

Reid et al. [9] reported a delayed tSCI diagnosis rate of 15% among patients with spinal injury, with an up to 8-fold increase in the incidence of permanent neurological deficit among patients with delayed diagnosis. An United States study reported that the incidence rate of delayed tSCI diagnosis among patients with cervical spinal injury was 4.6%, and 29% of these patients developed neurological sequelae because of the delay [10]. However, the two preceding reports were published before computed tomography (CT) scans were routinely performed for tSCI diagnosis. A 2002 study from the United Kingdom indicated that the incidence rate of delayed tSCI diagnosis among all tSCI patients was 9.1%, with half of these patients developing neurological deterioration because of mismanagement. The incidence of delayed diagnosis of cervical SCI has more than doubled during the past decade [11, 12]. Other studies have revealed variable incidence rates of delayed tSCI diagnosis, depending on the different definitions of the denominator used [13–15]. The main reasons for delayed tSCI diagnosis have been reported to be insufficiency of screening methods, errors in image interpretation, inadequacy of physical examination, and ambiguity in symptom presentation [9–11, 13]. However, such risk factors for delayed tSCI diagnosis have rarely been analyzed from a nationwide perspective [15].

In Taiwan, the national SCI incidence is 2.46 per 10000 person-years and trauma is the cause in 61% of such patients [16]. Among tSCI patients, injury at the cervical level, male sex and being older than 60 years were predominate characteristics [16]. In emergency departments (EDs) throughout Taiwan, trauma patients suspected of having tSCI are subjected to thorough neurological evaluation by a neurosurgeon or orthopedic surgeon. For diagnosis and further decision making plus management, a Magnetic Resonance Imaging (MRI) will also be done immediately. The International Standards for Neurological Classification of Spinal Cord Injury scale is commonly applied to assess and record the severity of neurological injuries [17]. In Taiwan, most tSCI patients are treated by neurosurgeons in moderate or severe emergent rescuer responsive hospitals that have sophisticated tSCI diagnosis and treatment capacities. Intensive care unit (ICU) admission depends on the SCI severity as well as associated injuries and vital signs. All tSCI patients undergo a rehabilitation program before hospital discharge. Over the 10 year period of the present study (2001–2011), no obvious change in tSCI care, such as megadose steroid therapy or MRI use was noted.

Rehospitalization is a frequently used indicator of the quality of care received during the first hospitalization [18]. Hence we used rehospitalization with new tSCI diagnosis as an indicator of the diagnostic quality of tSCI during the first hospitalization. Specifically, we conducted a nested case–control study of a nationwide population-based cohort to identify the risk factors for rehospitalization with new tSCI diagnosis. By using this indicator as a surrogate of delayed tSCI diagnosis, we estimated the incidence rate of and risk factors for delayed tSCI diagnosis. We also emphasized the key points for reducing the occurrence of delayed tSCI diagnosis among hospitalized trauma patients.

## Materials and methods

To secure patient confidentiality, the Ministry of Health and Welfare (MHW) strips the identifiable patient information from the National Health Insurance Research Database (NHIRD). Our study was exempted from approval by the Institutional Review Board of Taipei Medical University (No: 201311009).

The National Health Insurance (NHI) program of Taiwan is mandatory and covers nearly 99% of its population [16]. The NHIRD, provided by the NHI Administration, offers researchers the opportunity to use a nationwide cohort for investigating the concerns regarding tSCI [16, 19, 20].

**Fig 1:**
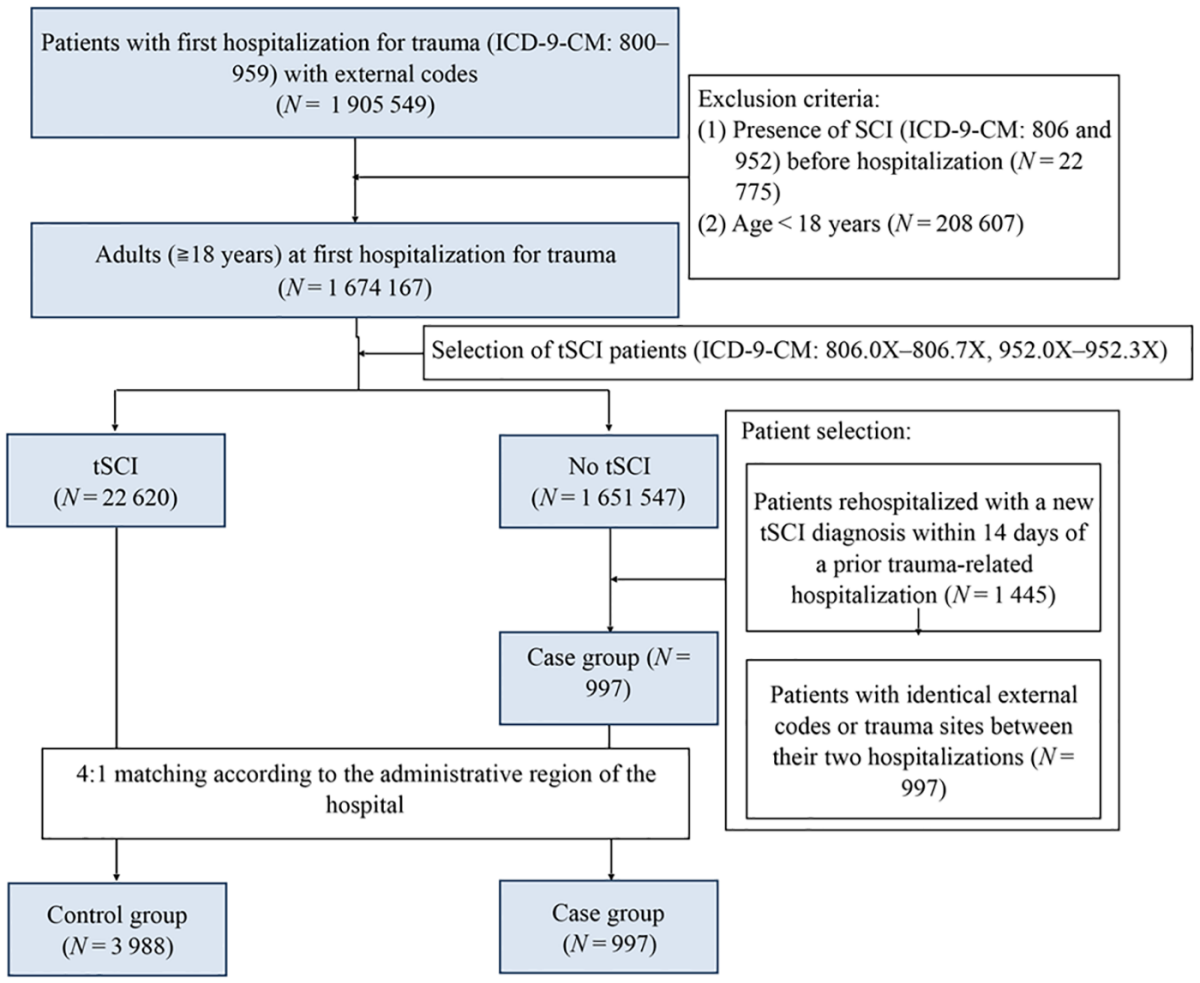
Patient selection from the Taiwan NHIRD between 2001 and 2011.

We selected patients who were first hospitalized for traumatic injury between 2001 and 2011, and only included those patients with both International Classification of Diseases, Ninth Revision, Clinical Modification (ICD-9-CM) codes for trauma (800–959) and a complete record of external codes. In our study, tSCI was defined using ICD-9-CM codes 806.0X–806.7X and 952.0X–952.3X. The exclusion criteria were (1) an age younger than 18 years and (2) presence of SCI (ICD-9-CM: 806 and 952) before hospitalization. The control group comprised patients diagnosed with tSCI at the first hospitalization, and the case group consisted of patients newly diagnosed with tSCI during rehospitalization within 14 days from the discharge of the first hospitalization (Fig 1). To strengthen the causality between the first hospitalization and rehospitalization in the case group, we included only patients with identical external codes or trauma sites between the two hospitalizations.

We selected 14 days as the duration between the first hospitalization and rehospitalization because a shorter interval provided a higher degree of confidence in causally associating the clinical course of the first hospitalization with that of the rehospitalization [21, 22]. The Medicaid Health Plan uses a similar 15-day interval to define rehospitalization for evaluating quality of care [23].

The candidate risk factors examined in our study were sex, age, Charlson comorbidity index [24], interhospital transfer, injury severity score (ISS), associated injury, admitted department, injury mechanism, and spinal injury level. Length of hospitalization (LOH) and spine abbreviated injury scale (AIS) [25] were not used as independent variables because they shared significant collinearity with the ISS (*r* = 0.20, *P*<0.0001 and *r* = 0.68, *P*<0.0001, respectively). We extracted all the variables from the patients’ first hospitalization records for the control group. For the case group, most of the variables were extracted from the patients’ first hospitalization records, except for the level of spinal injury and spine AIS scores, which were extracted from the rehospitalization records. Interhospital transfers were identified according to a code that described the patients’ condition at hospital discharge in the NHIRD. In Taiwan, the interhospital transfer of tSCI patients is arranged either by the treating physician due to inadequate capacities or facilities for continued treatment (e.g., tSCI patient treatment in a community hospital without MRI), or under the request of the patient/family members due to current treatment disapproval. We used the ICD Programs for Injury Categorization, which has previously been validated [26–28], to translate the ICD-9-CM codes into spine AIS scores and ISSs because the NHIRD does not record this clinical information. The estimated ISSs were classified as minor or moderate (<16), severe (16–24), or profound (>24) [29], whereas the estimated spine AIS scores were categorized into four groups, namely spine AIS 3, 4, 5, and 6 [25]. Associated injuries were those of the brain (ICD-9-CM: 800, 801, 803, and 850–854), chest (807 and 860–862), abdomen (863–866 and 868), face (802), pelvis (808 and 867), and extremities (810–829). Admitted departments were categorized as neurosurgery, orthopedic, and other surgical disciplines including general, chest, plastic, genitourinary, and cardiovascular surgery. The mechanisms of injury were classified according to the E-codes of the ICD-9-CM as motor vehicle collisions (E810–E819 and E958.5), falls (E880–E886, E888, and E957), and others. The injury levels were cervical (ICD-9-CM: 806.0–1 and 952.0), thoracic (806.2–3 and 952.1), lumbar (806.4–5 and 952.2), sacral (806.6–7 and 952.3), and multiple (i.e., injuries involving more than one level). Notably, sacral injury was excluded from the subsequent multivariate analysis because the numbers were insufficient.

Regional variation in health resources was controlled through 1:4 matching of the case and control groups according to the administrative region of the hospital; the NHI Administration of MHW divides the country into six administrative regions [30].

### Statistical analysis

We first performed a univariate analysis on the differences between the case and control groups. The Pearson chi-squared test was applied to assess categorical variables and the Cochran–Armitage trend test was used to assess ordered categorical variables. An independent *t* test was used for assessing the continuous variable. Stepwise selection method of logistic regression models was used to analyze the variables’ association with 14-day rehospitalization with new tSCI diagnosis. All exploratory variables were entered into the multiple logistic regression models. In our stepwise logistic regression analysis, the significance level for entry was 0.10, and the significance level for stay was 0.10. Odds ratios (ORs) and 95% confidence intervals (CIs) were applied to quantify the risk of rehospitalization with new tSCI diagnosis. Given the collinearity between the ISS and spine AIS scores (*r* = 0.68, *P*<0.0001), the spine AIS scores were stratified to cope with the interactions on the regression model. A two-sided *P* < 0.05 was considered to be statistically significant. All statistical analyses were performed using Statistical Analysis System (SAS) software, Version 9.3 (SAS Institute, Cary, NC, USA).

## Results

Among the 1 674 167 patients who were first hospitalized for traumatic injury between 2001 and 2011, 23 617 were diagnosed with tSCI. Of these patients, 997 were rehospitalized within 14 days with new tSCI diagnosis (case group) and 22 620 received a diagnosis of tSCI during their first hospitalization (control group). Thus, the incidence rate of 14-day rehospitalization with new tSCI diagnosis in our nationwide population-based cohort was 4.22%. After the case and control groups were matched at a 1:4 ratio, a total of 4985 patients were included in the analysis, with the control and case groups comprising 3988and 997 patients, respectively (Fig 1).

The univariate analysis results are presented in Table 1. We observed significant differences in data regarding the Charlson comorbidity index; interhospital transfers; ISSs; mean LOH; associated brain, chest, abdominal, pelvic, and extremity injuries; admitted departments; injury mechanisms; spine AIS; and spinal injury levels. Most patients in the case group (85%) had ISSs of less than 16. A negative trend was revealed between the ISSs and incidence of 14-day rehospitalization with new tSCI diagnosis (*P* < 0.0001).

**Table 1:** Demographic variables, comorbidities, and injury patterns in the control and case groups.

Variable	All patients				*P*	Patients with ISS < 16				*P*
	Control group		Case group			Control group		Case group		
	(*N* = 3988)		(*N* = 997)			(*N* = 2412)		(*N* = 848)		
	*n*	%	*n*	%		*n*	%	*n*	%	
Sex										
Female	1377	81.58	311	18.42	0.0389	886	75.79	283	24.21	0.0699
Male	2569	79.09	679	20.91		1502	72.88	559	27.12	
Missing ^a^	42	1.05	7	0.70		24	1.00	6	0.71	
Age (years)										
18–25	418	76.84	126	23.16	0.1982	280	72.54	106	27.46	0.2672
26–45	1200	80.16	297	19.84		764	75.64	246	24.36	
46–65	1397	79.97	350	20.03		795	72.27	305	27.73	
>65	973	81.29	224	18.71		573	75.00	191	25.00	
Charlson comorbidity index										
0	2963	79.14	781	20.86	0.0128	1772	72.77	663	27.23	0.0129
1	554	81.23	128	18.77		345	75.99	109	24.01	
>1	471	84.26	88	15.74		295	79.51	76	20.49	
Interhospital transfer										
No	3804	84.27	710	15.73	<0.0001	2319	79.12	612	20.88	<0.0001
Yes	184	39.07	287	60.93		93	28.27	236	71.73	
ISS										
<16	2412	73.99	848	26.01	<0.0001^d^	--	--	--	--	--
16–24	1332	92.05	115	7.95		--	--	--	--	
>24	243	88.36	32	11.64		--	--	--	--	
Mean LOH (SD) (days)	12.20	(16.80)	6.50	(12.24)	<0.0001^e^	10.26	(14.24)	5.86	(11.60)	<0.0001^e^
Brain injury										
No	2719	84.84	486	15.16	<0.0001	1692	78.88	453	21.12	<0.0001
Yes	1269	71.29	511	28.71		720	64.57	395	35.43	
Chest injury										
No	3803	80.86	900	19.14	<0.0001	2365	74.68	802	25.32	<0.0001
Yes	185	65.60	97	34.40		47	50.54	46	49.46	
Abdominal injury										
No	3934	80.32	964	19.68	0.0080	2392	74.24	830	25.76	0.0025
Yes	54	62.07	33	37.93		20	52.63	18	47.37	
Facial injury										
No	3892	80.28	956	19.72	0.0032	2370	74.27	821	25.73	0.0121
Yes	96	70.07	41	29.93		42	60.87	27	39.13	
Pelvic injury										
No	3947	80.35	965	19.65	<0.0001	2388	74.30	826	25.70	0.0007
Yes	41	56.16	32	43.84		24	52.17	22	47.83	
Extremity injury										
No	3620	81.26	835	18.74	<0.0001	2245	75.41	732	24.59	<0.0001
Yes	368	69.43	162	30.57		167	59.01	116	40.99	
Admitted department										
Neurosurgery	2334	87.12	345	12.88	<0.0001	1317	82.42	281	17.58	<0.0001
Orthopedics	626	71.62	248	28.38		470	67.63	225	32.37	
Other surgical disciplines^b^	1028	71.79	404	28.21		625	64.63	342	35.37	
Injury mechanism										
Motor vehicle collision	2127	76.18	665	23.82	<0.0001	1249	69.04	560	30.96	<0.0001
Fall	1263	82.50	268	17.50		756	76.36	234	23.64	
Other	598	90.33	64	9.67		407	88.29	54	11.71	
Spine abbreviated injury scale^c^										
AIS 3	2777	78.87	744	21.13	0.0132^d^	2410	79.20	633	20.80	<.0001^d^
AIS 4	1092	83.11	222	16.89		0	0.00	190	100.00	
AIS 5	69	75.82	22	24.18		0	0.00	17	100.00	
AIS 6	47	83.93	9	16.07		0	0.00	8	100.00	
Spinal injury level										
Cervical	2915	81.49	662	18.51	<0.0001	1644	74.39	566	25.61	<0.0001
Thoracic	315	73.09	116	26.91		182	67.16	89	32.84	
Lumbar	642	78.01	181	21.99		561	77.38	164	22.62	
Sacral	24	63.16	14	36.84		21	67.74	10	32.26	
Multiple	92	79.31	24	20.69		4	17.39	19	82.61	

^a^ Missing values in the table represent frequencies (column percentage).

^b^ Other surgical disciplines included general, chest, plastic, genitourinary, and cardiovascular surgery.

^c^The spine AIS scores were classified as contusion with transient neurologic deficit (AIS 3), incomplete cord syndrome (AIS 4), complete cord syndrome (AIS 5), or cord contusion at C3 or above (AIS 6).

^d^ Cochran–Armitage trend test.

^e^ *t* test.

AIS, abbreviated injury scale; ISS, injury severity score; LOH, length of hospitalization; SD, standard deviation.

Table 2 presents the results of the multiple logistic regression analysis. A significantly increased risk of 14-day rehospitalization with new tSCI diagnosis was evident in the patients with interhospital transfer (OR, 8.20; 95% CI, 6.48–10.38). The risk was significantly lower in patients with severe (ISS, 16–24; OR, 0.17; 95% CI, 0.13–0.21) and profound (ISS > 24; OR, 0.11; 95% CI, 0.07–0.18) injuries than in those with minor or moderate injury levels. Among the associated injuries, injuries in the brain (OR, 2.82; 95% CI: 2.36–3.37), chest (OR, 2.99; 95% CI, 2.14–4.19), and abdomen (OR, 2.74; 95% CI, 1.62–4.62) were risk factors. Compared with that in the neurosurgery department, hospitalization in the orthopedic department (OR, 2.26; 95% CI, 1.78–2.87) or in departments of other surgical disciplines (OR, 1.89; 95% CI, 1.57–2.28) were also risk factors. Compared with fall injuries, motor vehicle collision injuries were associated with a significantly increased risk of 14-day rehospitalization with new tSCI diagnosis (OR: 1.36; 95% CI: 1.12–1.65). Compared with injury at the cervical level, injuries at the thoracic (OR, 1.62; 95% CI, 1.21–2.18), lumbar (OR, 1.30; 95% CI, 1.02–1.65), and multiple (OR, 3.23; 95% CI, 1.86–5.61) levels were significant risk factors for 14-day rehospitalization with new tSCI diagnosis.

**Table 2:** Multiple logistic regression analysis of 14-day rehospitalization with new tSCI diagnosis (*N* = 4985).

Variable	OR	95% CI	*P*
Sex			
Female	1.00		
Male	1.30	1.09–1.54	0.0037
Interhospital transfer	8.20	6.48–10.38	<0.0001
ISS			
<16	1.00		
16–24	0.17	0.13–0.21	<0.0001
>24	0.11	0.07–0.18	<0.0001
Brain injury	2.82	2.36–3.37	<0.0001
Chest injury	2.99	2.14–4.19	<0.0001
Abdominal injury	2.74	1.62–4.62	0.0002
Facial injury	1.58	1.00–2.48	0.0483
Pelvic injury	1.84	1.02–3.32	0.0427
Extremity injury	1.52	1.19–1.96	0.001
Admitted department			
Neurosurgery	1.00		
Orthopedics	2.26	1.78–2.87	<0.0001
Other surgical disciplines^a^	1.89	1.57–2.28	<0.0001
Injury mechanism			
Fall	1.00		
Motor vehicle collision	1.36	1.12–1.65	0.0019
Other	0.45	0.32–0.62	<0.0001
Spinal injury level			
Cervical	1.00		
Thoracic	1.62	1.21–2.18	0.0012
Lumbar	1.30	1.02–1.65	0.0352
Multiple	3.23	1.86–5.61	<0.0001

^a^ Other surgical disciplines included general, chest, plastic, genitourinary, and cardiovascular surgery.

ISS, injury severity score; OR, odds ratio; CI, confidence interval.

Because 85% of patients in the case group had an ISS of <16, with a significantly higher risk of 14-day rehospitalization with new tSCI diagnosis, we performed a subgroup analysis on only these patients. We applied the same statistical methods used for the entire sample and a total of 3260 patients were examined. The results of the univariate analysis and multiple logistic regression analysis are presented in Tables 1 and 3, respectively. Overall, the risk of 14-day rehospitalization with new tSCI diagnosis remained significantly higher among these patients with interhospital transfer (OR, 8.79; 95% CI, 6.66–11.60); with an associated injury of the brain (OR, 2.18; 95% CI, 1.78–2.66), chest (OR, 1.91; 95% CI, 1.18–3.09), or abdomen(OR: 2.56; 95% CI: 1.27–5.14) and admitted to the departments of orthopedics (OR, 2.37; 95% CI, 1.83–3.06) or other surgical disciplines (OR, 2.02; 95% CI, 1.64–2.48). Motor vehicle collisions remained a significant risk factor for 14-day rehospitalization with new tSCI diagnosis (OR, 1.42; 95% CI, 1.15–1.76) as did injuries at the thoracic (OR, 1.54; 95% CI, 1.10–2.15) and multiple (OR, 18.30; 95% CI, 5.87–56.99) levels.

**Table 3:** Multiple logistic regression analysis of 14-day rehospitalization with new tSCI diagnosis for the patients with ISS < 16 (*N* = 3260).

Variable	OR	95% CI	*P*
Sex			
Female	1.00		
Male	1.18	0.98–1.43	0.0792
Interhospital transfer	8.79	6.66–11.60	<0.0001
Brain injury	2.18	1.78–2.66	<0.0001
Chest injury	1.91	1.18–3.09	0.0085
Abdominal injury	2.56	1.27–5.14	0.0084
Extremity injury	1.43	1.07–1.93	0.0171
Admitted department			
Neurosurgery	1.00		
Orthopedics	2.37	1.83–3.06	<0.0001
Other surgical disciplines^a^	2.02	1.64–2.48	<0.0001
Injury mechanism			
Fall	1.00		
Motor vehicle collision	1.42	1.15–1.76	0.0013
Other	0.40	0.28–0.57	<0.0001
Spinal injury level			
Cervical	1.00		
Thoracic	1.54	1.10–2.15	0.0124
Lumbar	1.13	0.87–1.47	0.3439
Multiple	18.30	5.87–56.99	<0.0001

^a^ Other surgical disciplines included general, chest, plastic, genitourinary, and cardiovascular surgery.

ISS, injury severity score; OR, odds ratio; CI, confidence interval.

Among the results of the spine AIS-stratified analysis, we only reported the results of the multiple logistic regression of spine AIS 3 (Table 4) because of the insufficient robustness of the AIS 4–6 models. The risk of 14-day rehospitalization with new tSCI diagnosis was significantly lower in patients with severe (ISS, 16–24; OR, 0.57; 95% CI, 0.41–0.79) and profound (ISS > 24; OR, 0.51; 95% CI, 0.29–0.92) injuries. The risk was significantly higher among patients with interhospital transfer (OR, 9.20; 95% CI, 7.03–12.04); with an associated injury of the brain (OR, 2.55; 95% CI, 2.07–3.14) or chest (OR, 1.83; 95% CI, 1.25–2.69); admitted to departments of orthopedics (OR, 2.19; 95% CI, 1.68–2.85) or other surgical disciplines (OR, 1.74; 95% CI, 1.40–2.15); with injuries at the thoracic (OR, 1.69; 95% CI, 1.20–2.38) or lumbar (OR, 1.67; 95% CI, 1.29–2.16) level. Motor vehicle collisions remained a significant risk factor (OR, 1.40; 95% CI, 1.12–1.74).

**Table 4:** Stratified analysis of 14-day rehospitalization with new tSCI diagnosis for the patients with spine AIS 3 (*N* = 3521).

Variable	OR	95% CI	*P*
Sex			
Female	1.00		
Male	1.27	1.04–1.54	0.0178
Interhospital transfer	9.20	7.03–12.04	<0.0001
ISS			
<16	1.00		
16–24	0.57	0.41–0.79	0.0008
>24	0.51	0.29–0.92	0.0244
Brain injury	2.55	2.07–3.14	<0.0001
Chest injury	1.83	1.25–2.69	0.0019
Abdominal injury	1.67	0.90–3.09	0.1039
Facial injury	1.51	0.90–2.54	0.116
Pelvic injury	1.21	0.62–2.35	0.5733
Extremity injury	1.18	0.89–1.57	0.2597
Admitted department			
Neurosurgery	1.00		
Orthopedics	2.19	1.68–2.85	<0.0001
Other surgical disciplines^a^	1.74	1.40–2.15	<0.0001
Injury mechanism			
Fall	1.00		
Motor vehicle collision	1.40	1.12–1.74	0.0026
Other	0.49	0.35–0.71	0.0001
Spinal injury level			
Cervical	1.00		
Thoracic	1.69	1.20–2.38	0.0025
Lumbar	1.67	1.29–2.16	<0.0001
Multiple	1.48	0.75–2.90	0.2562

^a^ Other surgical disciplines included general, chest, plastic, genitourinary, and cardiovascular surgery.

ISS, injury severity score; OR, odds ratio; CI, confidence interval.

## Discussion

Delayed tSCI diagnosis has been reported in patients with mild injuries [31, 32]. Traditionally, physiological parameters, such as paralysis, and injury mechanisms, such as high-energy impact, are crucial for alerting surgeons about tSCI [33]. Therefore, the absence of typical neurological impairments or suspicious injury mechanisms may prevent or delay tSCI diagnosis within a reasonable timeline. Bicknell et al. [31] indicated that most cases of delayed tSCI diagnosis were due to incomplete cord injury. Schneider et al. [34] reported that patients with pathological narrowing of the spinal canals may experience central cord injury because of a hyperextension injury resulting from slight trauma. In Australia, tSCI patients were identified as being more likely to face a delay in receiving definitive treatment; that study confirmed that a lack of significant injury mechanisms or neurological symptoms led to failure of diagnosis [35]. Our study demonstrated that compared with severe or profound injuries, an injury with ISS <16 is a significant risk factor for 14-day rehospitalization with new tSCI diagnosis. Our case group patients mostly sustained mild or moderate injuries during their first hospitalizations and were discharged or transferred from the hospital of initial treatment without tSCI diagnosis. Treating surgeons may easily overlook tSCIs if neurological deficits are subtle. By contrast, patients with more serious injuries generally receive a more meticulous evaluation during hospitalization, with their tSCIs likely to be identified before discharge. In our study, the mean LOH was 6.5 days in the case group, whereas that of the control group was 12.2 days.

**Fig 2:**
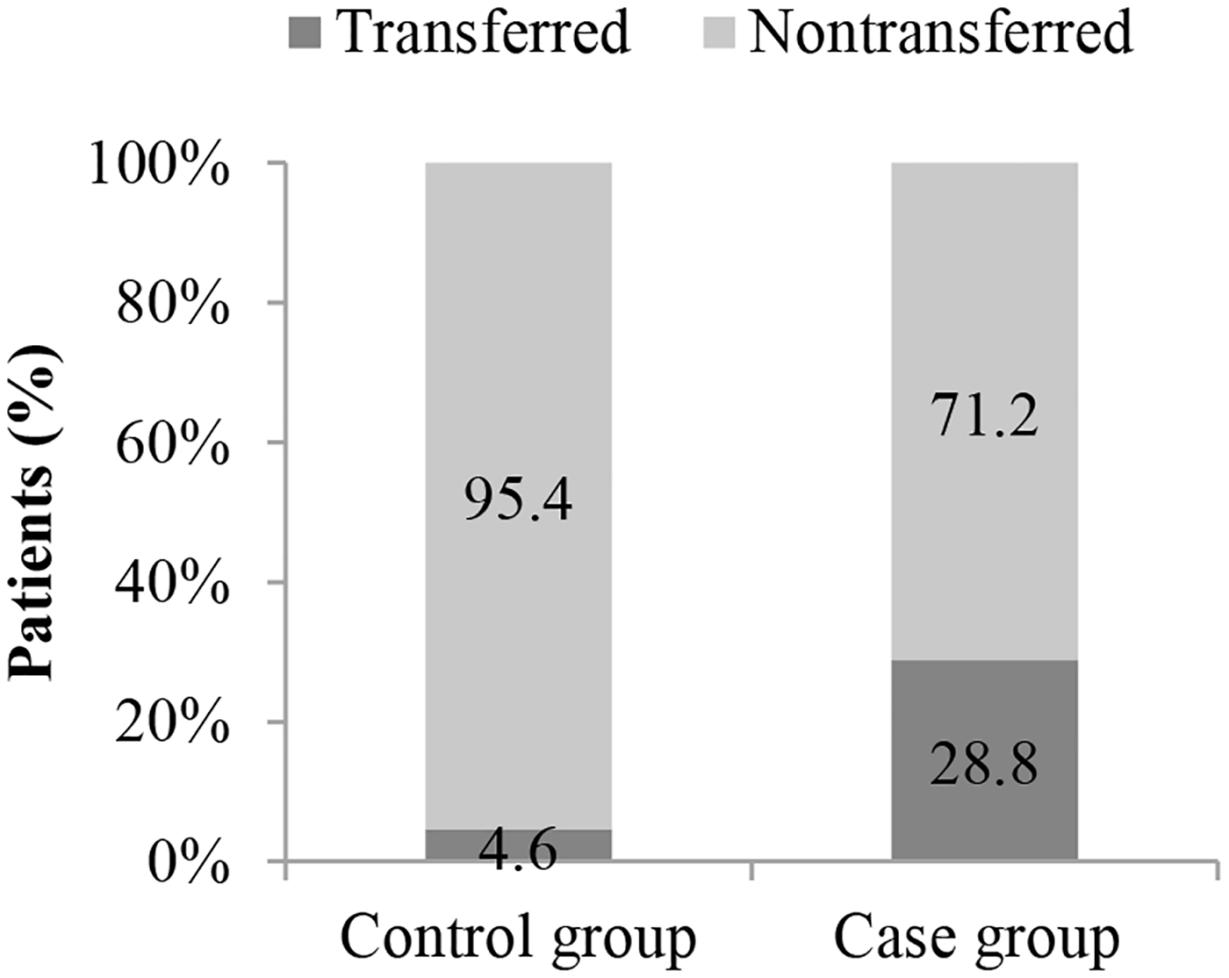
Transfer rates in the case (N = 997) and control (N = 3988) groups.

**Fig 3:**
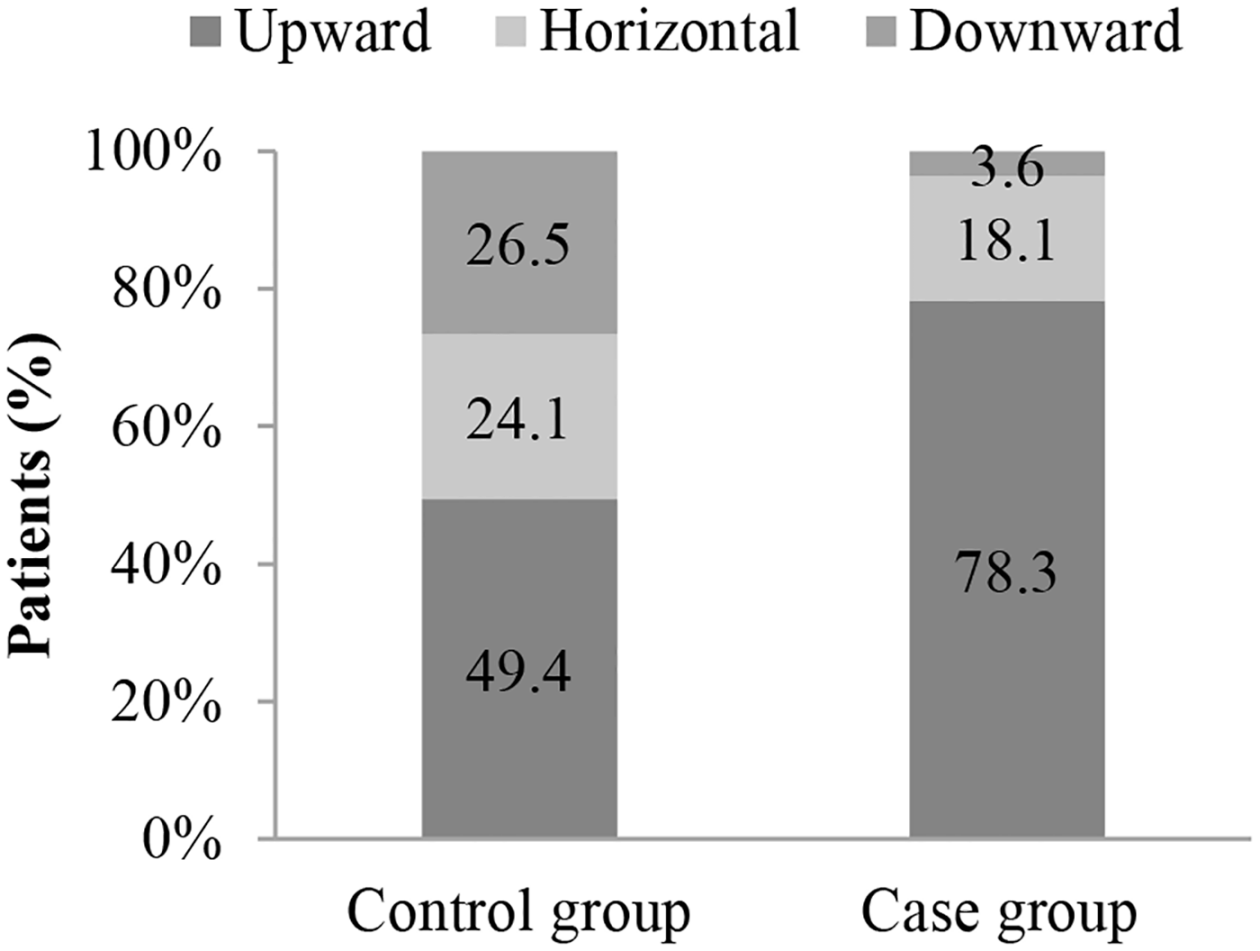
Transfer patterns in the case (N = 287) and control (N = 184) groups.

Patients with ISSs < 16 may not fulfill the trauma center criteria and be transported to nearby hospitals that do not have the appropriate resources for diagnosing tSCI. Interestingly, our study also demonstrated that interhospital transfer is a highly significant risk factor for 14-day rehospitalization with new tSCI diagnosis (OR, 8.20). Only 4.6% of our control group patients underwent an interhospital transfer; 49.4% of these patients were transferred from a lower-level to a higher-level hospital. All transferred patients in the control group obtained tSCI diagnoses before leaving the transferring hospitals. By contrast, 28.8% of our case group patients underwent an interhospital transfer during their first hospitalizations; 78.3% of these patients were transferred from lower-level to higher-level hospitals. In contrast to the transferred patients in the control group, no diagnosis of tSCI was made for the transferred patients in the case group before they left the transferring hospitals (Figs 2 and 3).

Middleton et al. [35] reported that 60% of tSCI patients underwent multiple transfers before receiving definitive treatment. A study in Australia identified rural hospitals as the major source of interhospital transfers among tSCI patients, and 18% of these transfers were only made for the exclusion of tSCI [36]. Our study demonstrated that trauma patients treated in lower-level hospitals were more likely to have interhospital transfers without a diagnosis of tSCI. Lower-level hospitals are generally community based and lack diagnostic facilities or subspecialists for the management of complex neurotraumas, whereas higher-level hospitals are usually medical centers, which can provide advanced resources, such as MRI equipment and experienced surgeons who can diagnose occult tSCI.

The transferred patients in the case group were at a high risk of neurological deterioration because their tSCI was not identified before the transfer [37]. Therefore, acute care providers at receiving hospitals should search for occult signs of tSCI in transferred trauma patients, particularly in those with ISSs < 16 or those transferred from lower-level hospitals.

We hypothesized that trauma patients treated by surgeons familiar with neurotrauma have a reduced risk of 14-day rehospitalization with new tSCI diagnosis. Therefore, we compared this risk among the admitted departments between the case and control groups. We observed that compared with that in a neurosurgical department, hospitalization in an orthopedic department (OR, 2.26) or those of other surgical disciplines (OR, 1.89) was risk factor for rehospitalization with new tSCI diagnosis. As expected, the risk was higher among the patients treated in orthopedic department since majority of these patients were admitted from the ED with extremity injury, which had been identified as risk factor (OR, 1.52) in our study. We also recommend that all surgeons involved in trauma care be cognizant of occult tSCI signs in their patients.

In our study injuries at the thoracic and lumbar levels were significant risk factors for rehospitalization with new tSCI diagnosis, although the risk for injuries at the lumbar level became insignificant among patients with ISS < 16 after stratification of ISS. Thoracolumbar (TL) spinal injury has an incidence rate of 5% in all blunt trauma patients and can occur in more than half of all patients presenting to hospitals with spinal injury [38, 39], almost 50% of these patients also exhibit neurological deficits [39–41]. In one study, delayed TL spinal injury diagnosis was found to increase the incidence of neurological deficits from 1.4% to 10.5% [9]. However, the evidence-based decision rules facilitating the diagnosis of TL spinal injury remain controversial [4, 38, 39, 42]. For instance, the guidelines published by the Eastern Association for the Surgery of Trauma recommend solely using clinical examination to exclude TL spinal injury for alert patients without a significant injury mechanism [39]. However, Anderson et al. [38] mentioned that almost 14% of TL spinal injury patients were awake and had normal results of back examination. In addition, the sensitivity of clinical examination alone in detecting TL fractures requiring surgery among evaluable patients is poor (88.3%) [4]. We believe that further validation and implementation of evidence-based decision rules will improve the diagnostic quality of TL tSCI.

Because the spinal cord is well protected by strong muscles and bony structures, tSCI is a rare condition, with a global incidence rate of 23 tSCI cases per 1 million people in 2007 [43]. New injury cases resulting in tSCI occurring within 14 days after the first hospitalization did not account for an appreciable proportion of the patients included in our case group. Furthermore, our case group only included patients who were rehospitalized with the same external codes or trauma sites. These criteria should have substantially minimized the probability of including new tSCI occurring within 14 days after the first hospitalization.

Posttraumatic cervical instability may cause delayed presentation of tSCI in patients without obvious skeletal abnormality during their initial evaluation [44, 45]. For example, Delfini et al. [44] analyzed a series of 172 patients receiving delayed surgical treatment for their tSCI and demonstrated that 15% of these patients had cervical instability. Overall, SCI without a bony fracture is rare, with an incidence of only 0.7% [15]. Therefore, the influence of this injury type on the results of our study was minimal.

Miscoding is another possible factor causing erroneous recording of rehospitalization with new tSCI diagnosis. The NHIRD, which we used for identifying tSCI patients, is a database of insurance payment claims by medical care facilities. The tSCI is listed in the catastrophic illness registry and receives high reimbursement; therefore, the possibility of miscoding during the first hospitalization is unlikely. In addition, all cases of 14-day rehospitalization are carefully reviewed for care quality by the NHI Administration [46]. Because tSCI is a rare but catastrophic illness with high social impact, new tSCI diagnosis was unlikely to be due to miscoding during the 14-day rehospitalization.

We found that the results of the spine AIS-stratified analysis in the smaller subgroups with more severe tSCI (AIS 4–6) were unstable, and these subgroups’ effects cannot robustly be shown due to the technical limitation. On the contrary, our results can robustly demonstrated in patients with transient and mild neurological deficits (AIS 3). These patients formed the largest subgroup of our study and might suffer most from deterioration. We believe that this is an important finding.

Clinical data, such as physical or neurological examination records, images, and laboratory test results, are unavailable in the NHIRD. Therefore, the information required to explore the reason for delayed tSCI diagnosis was lacking in our study. Obtaining additional clinical data to describe the characteristics of tSCI patients at a high risk of delayed diagnosis and the underlying reasons for that risk in future studies will help target clinical education to ensure that tSCI patients receive correct and timely diagnoses.

A few other limitations with this study should be addressed. First, restricting the sample to first-time hospitalized trauma patients led to the exclusion of tSCI patients sustained in subsequent traumas within the study period (2001–2011). We also considered the lack of a previous validation of the coding quality of tSCI within the NHIRD to be a limitation of our study. Our study followed the Strengthening the Reporting of Observational Studies in Epidemiology guidelines. Finally, the NHIRD, which is representative of Taiwan, provides an opportunity for the realistic investigation of delayed diagnosis of tSCI. However, because of the differences in health care systems and SCI classification, our results may not be extrapolated to other countries.

## Conclusions

Fourteen-day rehospitalization with new tSCI diagnosis can be used as an indicator for the diagnostic quality of the first hospitalization and as a surrogate for delayed tSCI diagnosis. Delayed tSCI diagnosis is not uncommon, particularly among trauma patients with ISSs < 16 or those transferred from lower-level hospitals. All surgeons involved in trauma care should search for occult tSCI among their patients, particularly those with brain, chest or extremity injuries. Finally, we recommend further validation and implementation of evidence-based decision rules, which are essential for improving the diagnostic quality of TL tSCI.
